# Effect of concurrent training on physical performance and quality of life in children with malignancy: A systematic review and meta-analysis

**DOI:** 10.3389/fpubh.2023.1127255

**Published:** 2023-03-17

**Authors:** Wanli Zang, Mingqing Fang, Haohao Chen, Xinmeng Huang, Dong Li, Jin Yan, Heng Shu, Mingyuan Zhao

**Affiliations:** ^1^Postgraduate School, University of Harbin Sport, Harbin, China; ^2^Xiangya Hospital, Central South University, Changsha, China; ^3^Norman Bethune Health Science Center, Jilin University, Changchun, China; ^4^Department of International Culture Education, Chodang University, Muan-gun, Republic of Korea; ^5^Centre for Active Living and Learning, University of Newcastle, Callaghan, NSW, Australia; ^6^College of Human and Social Futures, University of Newcastle, Callaghan, NSW, Australia; ^7^Winter Olympic School, Harbin Sport University, Harbin, China

**Keywords:** pediatric, physical performance, quality of life, concurrent training, exercises (physical-skill)

## Abstract

**Objective:**

This study aims to evaluate the intervention effect of concurrent training on children with malignant tumors to provide evidence for prescribing exercise for children with malignant tumors.

**Methods:**

Twelve databases were searched from inception to October 15, 2022. Two researchers independently screened the literature, evaluated the quality, extracted the data, and performed the meta-analysis using R.

**Result:**

A total of nine randomized controlled trials involving 371 children were included in this study. The meta-analysis revealed that muscle strength was significantly greater in the exercise group compared to the usual care group [SMD = 0.26, 95% CI (0.04, 0.48), *P* = 0.023], with subgroup analysis showing no significant difference in upper limb [SMD = 0.13, 95% CI (−0.17, 0.43), *P* = 0.318] and a considerable difference in lower limb strength [SMD = 0.41, 95% CI (0.08, 0.74), *P* = 0.015]. Physical activity [SMD = 0.57, 95% CI (0.03, 1.1), *P* = 0.038], timed up and down stairs test [SMD = −1.22, 95% CI (−2.04, −0.4), *P* = 0.004], 6-min walking ability [SMD = 0.75, 95% CI (0.38, 1.11), *P* < 0.01], quality of life [SMD = 0.28, 95% CI (0.02, 0.53), *P* = 0.033], and cancer-related fatigue [SMD = −0.53, 95% CI (−0.86, −0.19), *P* = 0.002] were significantly better than the usual care group. There were no significant differences in peak oxygen uptake [SMD = 0.13, 95% CI (−0.18, 0.44), *P* = 0.397], depression [SMD = 0.06, 95% CI (−0.38, 0.5), *P* = 0.791], and withdrawal rates [RR = 0.59, 95% CI (0.21, 1.63), *P* = 0.308] between the two groups.

**Conclusion:**

Concurrent training could improve physical performance for children with malignancy but had no significant effect on mental health. Because the quality level of evidence is mostly very low, future high-quality randomized controlled trials are required to confirm these findings.

**Systematic review registration:**

https://www.crd.york.ac.uk/PROSPERO/display_record.php?RecordID=364140, identifier CRD42022308176.

## Introduction

Pediatric cancer is a significant cause of the global burden of childhood disease, with annual increases in incidence rates (1–3). Globally, an estimated 300,000 children and adolescents between 0 and 19 are diagnosed with cancer yearly (4), of which more than 175,000 (70%) are children under the age of 15. Today, tremendous advancements in cancer treatment have improved the survival rates of children and adolescents with cancer, with 5-year survival rates in developed countries exceeding 80% (5). Although the prognosis for children with cancer has improved significantly recently, the rate of improvement has slowed as the intensity of treatment for many pediatric cancers may have reached limitations (6).

The prevalence of severe chronic health conditions among long-term survivors of pediatric cancers is three times higher than in matched controls (7), with approximately two-thirds of pediatric cancer patients experiencing at least one chronic or long-term side effect after treatment (8). Side effects are health complications caused by cancer or cancer treatments (surgery, chemotherapy, radiation therapy, and stem cell transplantation) that do not disappear or recur months or years after treatment is completed, negatively impacting the quality of life and overall health (9). These effects include decreased muscle and soft tissue function (e.g., muscle weakness, radiation fibrosis syndrome, and soft tissue contractures), pain (10), peripheral neuropathy (11), fatigue, weakness (12), anxiety (13), range of motion limitations, and impairments such as balance and gait deficits (14), all of which can negatively affect a child's athletic performance (15) and quality of life (16, 17). Consequently, there is a growing awareness of the necessity for planned survivorship care, including medical follow-up and monitoring of the long-term effects of cancer treatment (18).

The benefits of aerobic and resistance training alone are well documented, but studies of concurrent exercise in pediatric populations are limited. Concurrent training improves the physical fitness and athletic performance of adolescents more than unimodal training (19) and refers to a combination of resistance and aerobic training to gain strength, muscle hypertrophy, explosive power, and endurance-enhancing training in the same training phase (20). Indeed, compared to single aerobic or resistance exercise, concurrent training is more effective in improving patients' cardiopulmonary function and muscle strength and has been used to rehabilitate adult patients (21) with chronic kidney disease (22), obesity (23), and cancer (24). However, due to anthropometric, physiological, and biomechanical differences, the characteristics of neuromuscular adaptation to concurrent training in children may differ from those of relatively older populations. The concurrent training program is complex, and the interaction of variables (e.g., training mode and intensity, training content, subject characteristics, training muscle groups, etc.) may induce different adaptations to train. Therefore, this study involved a meta-analysis to systematically evaluate the effects of concurrent training on exercise performance and quality of life in children with cancer to provide evidence for clinical healthcare professionals to prescribe exercise interventions in children with malignant tumors.

## Methods

This meta-analysis was performed and reported in accordance with PRISMA guidelines (The details are shown in Supplementary Appendix A) and registered with the PROSPERO (CRD42022308176).

### Literature search

We performed a systematic search for relevant articles written in English identified by title and abstract in OVID, PubMed, Web of Science, Embase, Cochrane Library, Scopus, SPORT Discus, CINAHL, SinoMed, Wanfang, CNKI, VIP from inception of the database to October 15, 2022. The search strategy was a combination of subject terms and free words (The details are shown in Supplementary Appendix B). In addition to the electronic literature search, additional searches were completed by examining reference lists of relevant articles to identify studies not retrieved from the databases.

### Study selection

First, duplicates were removed from the retrieved literature, then the titles and abstracts of the remaining articles were independently screened by two authors based on the inclusion and exclusion criteria. Manual screening of the references was also performed to identify further relevant studies. Finally, the full text of the conforming literature was obtained to decide whether to include it or not. Any differences in literature selection were discussed by the two authors to reach a consensus; otherwise, the decision was made by the third author. The inclusion criteria were as follows: (1) randomized controlled trials; (2) studies comparing concurrent training AND usual care as the control group to treat patients with pediatric cancer;(3) studies in which any of the following clinical outcomes were reported cancer-related fatigue, quality of life, 6-min walking ability (6WMT), Peak oxygen uptake (VO_2_peak), muscle strength, physical activity, timed up and down stair test, or depression. The exclusion criteria were: (1) non-clinical studies such as animal studies; (2) non-English and Chinese literature; (3) the full text of the literature was not available.

### Data extraction

The two authors extracted the data from the included studies, including: (1) the basic study such as title, first author, year of publication, cancer type, and disease stage; (2) experimental information such as study type, intervention method of the exercise group and control group; (3) patient information such as the number of patients in each group, gender ratio, age; (4) outcomes mentioned above.

### Quality assessment

The risk of bias in the RCTs was assessed in the domains of the randomization process, deviations from intended interventions, missing outcome data, measurement of the outcome, and selection of the reported result using the revised Cochrane risk of bias tool for randomized trials (RoB2). Any disagreement was negotiated between the two authors to reach a consensus, otherwise was decided by the third author. Each domain was rated as “low,” “some concerns,” or “high” and the overall ROB for each trial was based on the highest risk attributed to any one domain. The quality of the evidence for our outcomes was assessed using the Grading of Recommendations, Assessment, Development, and Evaluations (GRADE) framework.

### Statistical analysis

A meta-analysis of studies reporting results of physical capacity-related endpoints as “mean” (plus standard deviation (SD) or standard error of the mean) values before and after the exercise intervention was performed. For these continuous variables, pooled standardized mean differences of endpoint data across interventions (last evaluation at the end of the follow-up minus baseline) were computed. *I*^2^ was used to test for heterogeneity between studies, and the *P*-value was calculated. According to Cochrane's handbook, when *I*^2^ is more than 50%, the fixed effect model is used; otherwise, the random effect model. All data analyses were performed using the meta package in R (4.1.3) (25). However, due to the limited number of included studies, we did not test for publication bias.

### GRADE quality of meta evidence

The (GRADE) guidance tools were used to assess the quality of evidence (26). The GRADE system assesses five domains: study risk of bias, publication bias, indirectness, imprecision, and inconsistency, and assigns grading levels of high, moderate, low, and very low for each outcome.

## Results

### Literature search

**Figure 1:**
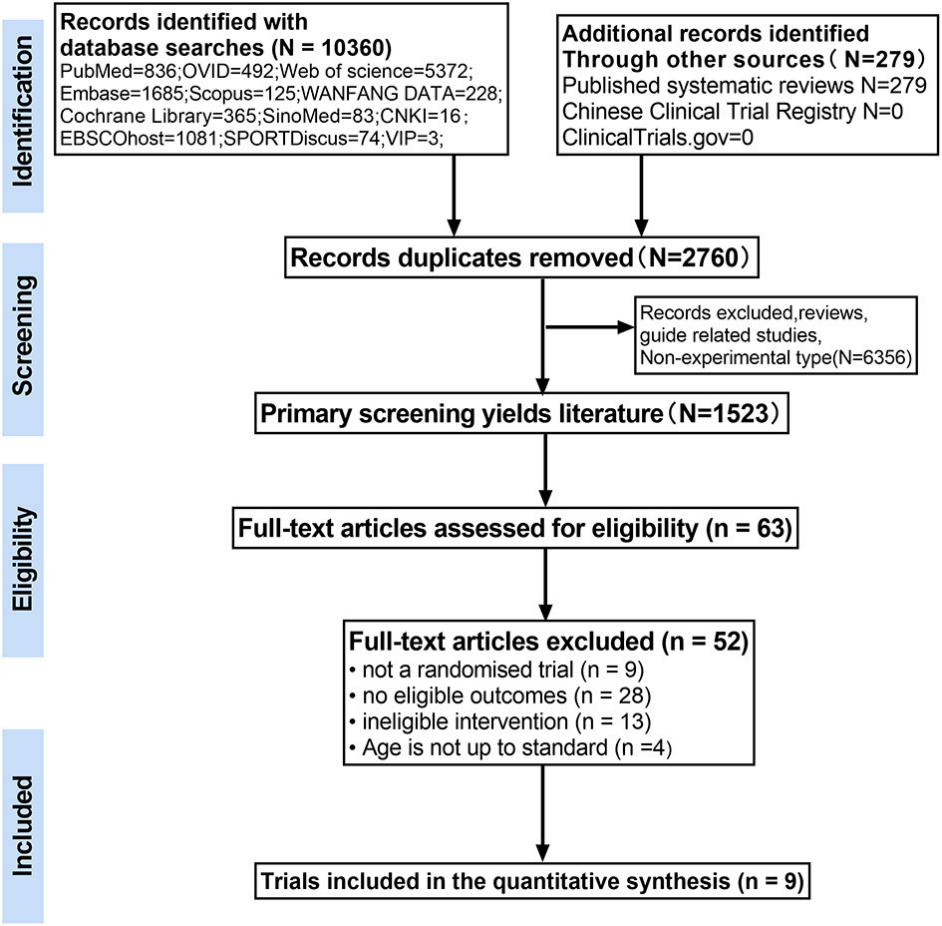
PRISMA flow diagram chart for systematic review and meta-analysis.

A total of 10,639 relevant articles were obtained for the initial review, and 63 potentially eligible articles were selected for full-text review. Of these, 54 articles were excluded for reasons including non-randomized controlled studies (*n* = 9), studies that included subjects who did not meet the inclusion criteria (*n* = 4), studies with interventions that did not meet the inclusion criteria (*n* = 13), and studies that did not have outcomes of interest (*n* = 28). A total of nine papers met the inclusion criteria and were used for further analysis (the flow chart of study selection is shown in Figure 1).

### Study characteristics

A total of nine trials involving 371 children with malignancies during or after treatment were analyzed in the meta-analysis. Only one of these studies was conducted in a developing country, and the others were conducted in developed nations or regions. The age of the study participants ranged from 4 to 18 years. The form of the concurrent training interventions varied among the studies, and the duration of the interventions for children with malignancy ranged from 6 to 24 weeks. The duration of one study was the entire hospitalization period, one study had an intervention duration of 6–8 weeks, and the remaining seven studies had a duration of 12–24 weeks. Six studies involved three interventions per week, and three studies had two interventions weekly. Nine studies had a duration of 60 mins or less per intervention. VO_2_ Peak, muscle strength, physical activity, walking ability, up and down stairs test, cancer-related fatigue, depression, and quality of life were used as outcome indicators. Data were pooled from the above studies and included in the meta-analysis. Detailed characteristics of the included studies are displayed in Table 1.

**Table 1:** The baseline characteristics of included trials.

**Study**	**County**	**Croup (*n*)**	**Age (years)**	**Conditions of participation**	**Interventions**	**Location**	**Exit (*n*)**	**Outcomes**
Braam et al. (27)	Netherlands	26	13.4 ± 3.1	Between 8 and 18 years of age. Assessed by an oncologist to be able to participate in physical activity.	Aerobic exercise + moderate intensity resistance exercise, 30min/time, 2 times/week for 12 weeks. Exercise intensity: from January to March, the intensity increased sequentially. The child's peak heart rate increased from 66 to 100%.	Hospital	1	① ② ③ ④ ⑤ ⑥ ⑦
		33	13.1 ± 3.1		The control group received usual care according to local guidelines and preferences.		2	⑧ ⑨
Manchola-González et al. (28)	Spain	12	11.8 ± 4.38	Between 7 and 17 years of age. Participants had complete remission of oncologic disease for at least 1 year and had completed chemotherapy at the time of starting the intervention program.	Warm-up + resistance exercise + aerobic exercise, 3 times/week for 16 weeks. The load was increased every 4 weeks; the exercise intensity was increased from 50 to 80% of the maximum exercise heart rate, and the resistance exercise was increased from 2 sets × 8 reps to 4 sets × 12 reps.	Home	0	① ② ④ ⑤
		7	11.0 ± 3.7		Maintain routine care.		3	⑨
Fiuza-Luces et al. (29)	Spain	16	10 ± 1	Between 4 and 18 years of age; new diagnosis of extracranial solid tumor; no previous treatment other than surgery; good performance status.	30 min of aerobic exercise (e.g., jogging, cycle ergometer, etc.) + 30 min of resistance exercise (e.g., dumbbell lifting, rowing exercises) with 1–2 min of rest in between, 3 times/week, (19 ± 2) weeks. The exercise intensity was 60 to 70% of the maximum heart rate.	Hospital	0	① ② ④ ⑥
		17	11 ± 1		Maintain routine care.		0	
Senn-Malashonak et al. (30)	Germany	28	11 (5–7)	(1) Age ≥ 5 years; (2) no cardiopulmonary, orthopedic, or neurological disorders; (3) no cardiac, pulmonary, orthopedic, or neurological disorders; and (4) no language impairment.	10–30 min of endurance training (Cycle ergometer, Aerobic variations, Sportive games) + Resistance exercise (3–5 exercises, 1–3 sets, 7–15 repetitions) + Flexibility training (3–5 exercises), 45–60 min/time, 3 times/week, exercise intervention continued throughout the hospitalization.	Hospital	0	② ③
		29	12 (6–8)		Receive routine hospital care + Mental training (10–30 min)+ Relaxation training.		0	
Stössel et al. (31)	Germany	14	10.6 ± 5.19	Patients are eligible if they are at least 4 years old and are treated with chemotherapy or radiation therapy.	15–20 min of aerobic exercise (such as walking, ball games) + 6–10 min of resistance exercise (with mini dumbbells, elastic bands, or body weight), 5–10 min of stretching before and after exercise, 45–60 min each time, 3 times/week, 6–8 weeks. The exercise intensity was 60–75% of the maximum heart rate. a score of12 to 13 on the Borg scale RPE6-20.	Hospital + home	2	① ③ ⑤ ⑥ ⑦ ⑨
		15	11.4 ± 4.25		Maintain routine care.		0	
Lam et al. (32)	Hong Kong	37	12.8 ± 2.5	9–18 years old; currently receiving active treatment. Children with evidence of recurrence or second malignancy, and children with physical impairment or cognitive and learning problems (identified from medical records) were excluded.	Aerobic + resistance exercise with post-exercise stretching and relaxation, from low to moderate intensity activity, for the first 4 weeks (2 sessions/week) and the next 20 weeks (1 session/week), for a total of 24 weeks.	Home	1	① ⑤ ⑥
		33	12.5 ± 2.5		The same 15-min health education lecture as in the experimental group. Placebo intervention activities included playing a card or chess game and providing health advice on flu prevention and the importance of a healthy diet.		2	⑦ ⑨
Marchese et al. (33)	USA	13	7.6	Children aged 4–18 years receiving maintenance treatment were eligible to participate in this study.	The program consisted of bilateral ankle dorsiflexion stretches lasting 30 s, 3 times per week, 5 days per week. Three sets of ten repetitions of bilateral lower extremity strengthening with daily aerobic fitness for 4 months.	Home	–	① ④ ⑥
		15	8.6		The control group did not receive physical health-related instructions or any physical therapy interventions.		–	
Dubnov-raz et al. (34)	Israel	10	11.1	Males and females aged 7–14 years who had received cancer treatment or bone marrow transplantation in the past and had completed all immunosuppressive treatments at least 6 months prior to study entry.	The main component consists of a 30 min strength and endurance workout in the gym using bands, balls, games, weights and various exercise machines. Activities ended with a 10–15 min cool down with gentler activities such as walking, abdominal crunches and stretching. 2 sessions per week for 6 months	Home	0	② ⑥ ⑧
		12	11.8		Maintain routine care.		0	
Tanir et al. (35)	Turkey	19	8–12	Between 8 and 12 years of age. In remission; not participating in a regular exercise program in the past 6 months; no previous history of cancer; no history of neurological or genetic disorders prior to diagnosis; no heart, lung, kidney or liver dysfunction; no problems with vision, hearing or perception.	Exercises include range of motion (ROM), leg muscle strengthening and aerobic exercise. 3 times a week. For a period of 3 months.	Hospital + home	0	③ ⑤ ⑤
		21			Maintain routine care.		0	

①: Muscle strength; ②: VO_2_Peak; ③: 6-min walking ability; ④: TUDS; ⑤: Physical activity; ⑥: Quality of life; ⑦: Cancer-related fatigue; ⑧: Depression; ⑨: Withdrawal rates.

### Quality assessment

**Figure 2:**
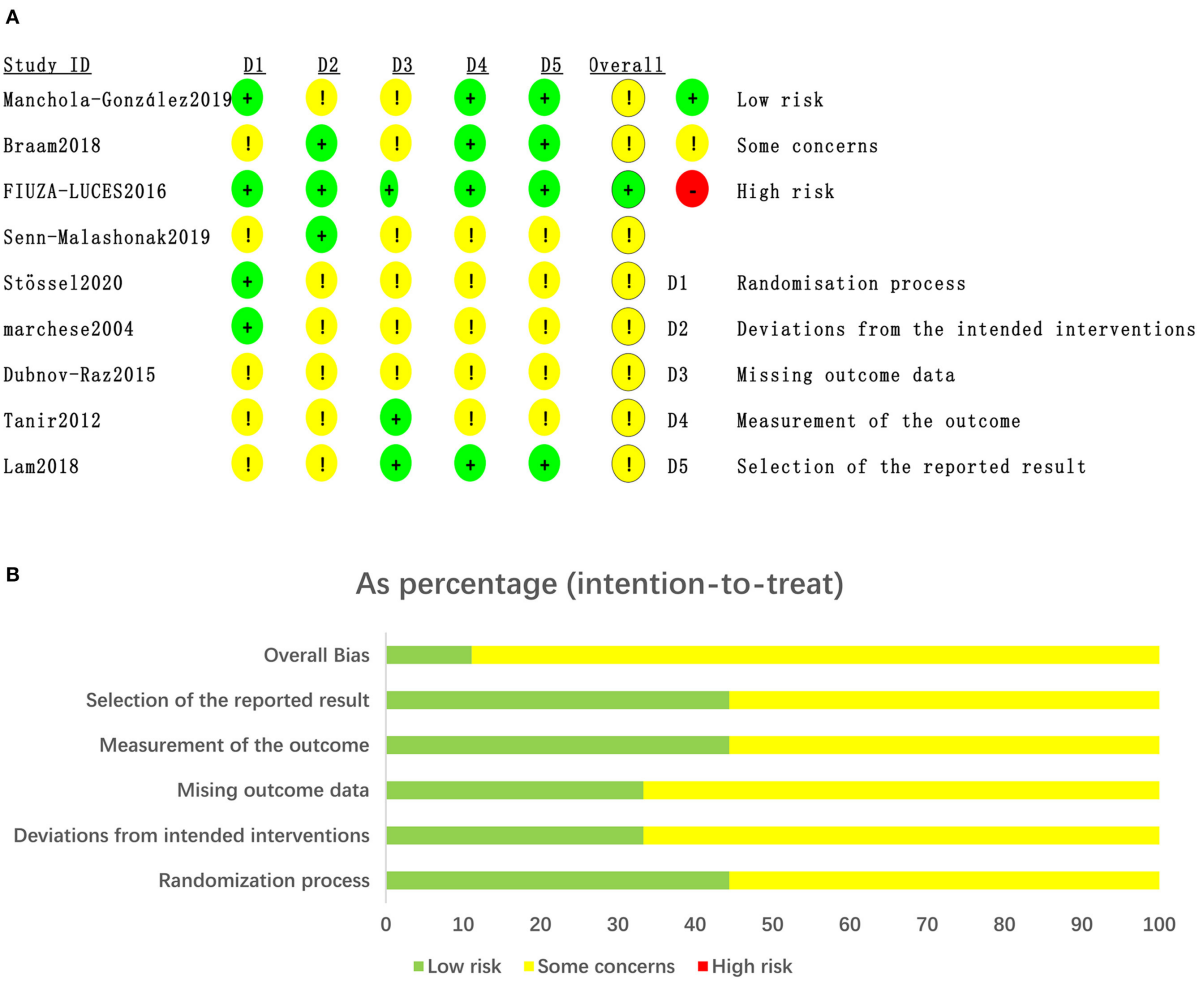
Quality of studies included in the meta-analysis. **(A)** Risk of bias graph and summary for the including 11RCTs. **(B)** The distribution of the methodological quality of included 11RCTs.

The risk of bias assessment for the included studies is illustrated in Figure 2.

### Muscle strength

**Figure 3:**
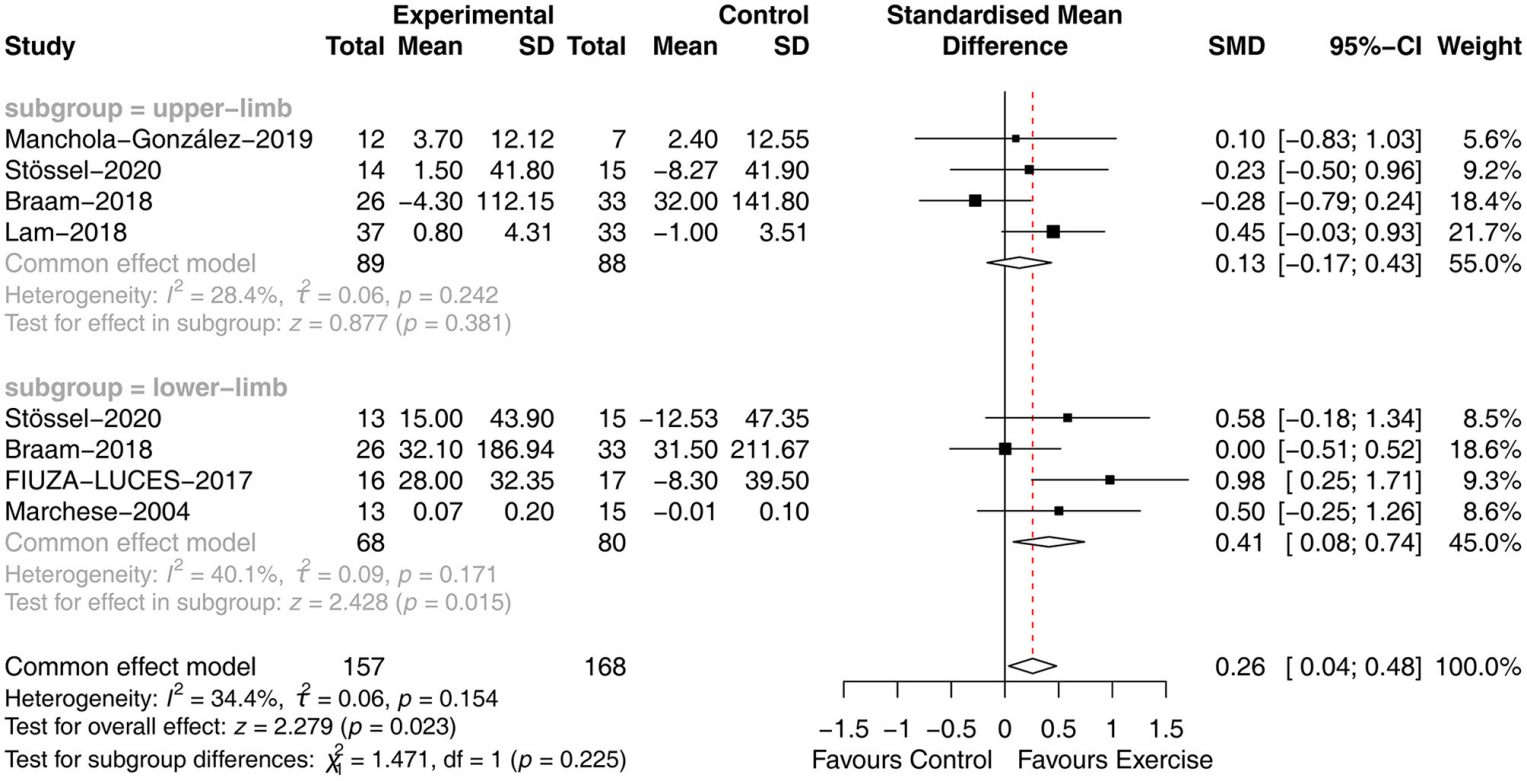
Results of meta-analysis of muscle strength.

Six studies (27, 29, 31–33, 36) analyzed the effect of concurrent training on muscle strength in children with malignancy. Due to the different measurement tools used, the SMD was used. The heterogeneity between studies was small (*P* = 0.154, *I*^2^ = 34.4%), and a fixed-effects model was used for analysis, showing that muscle strength was significantly improved in the exercise group compared to the usual care group [SMD = 0.26, 95% CI (0.04, 0.48), *P* = 0.023]. Four studies tested the upper extremity strength of children, and four studies tested the lower extremity strength of children. Subgroup analysis was performed using a fixed effects model, with less heterogeneity between studies for upper extremity strength (*P* = 0.242, *I*^2^ = 28.4%) and lower extremity strength (*P* = 0.171, *I*^2^ = 40.1%). However, subgroup analysis revealed differences in the effect of concurrent training on the impact of upper [SMD = 0.13, 95% CI (−0.17, 0.43), *P* = 0.318] and lower [SMD = 0.41, 95% CI (0.08, 0.74), *P* = 0.015] extremity strength in children with malignancy (Figure 3).

### VO_2_Peak

**Figure 4:**
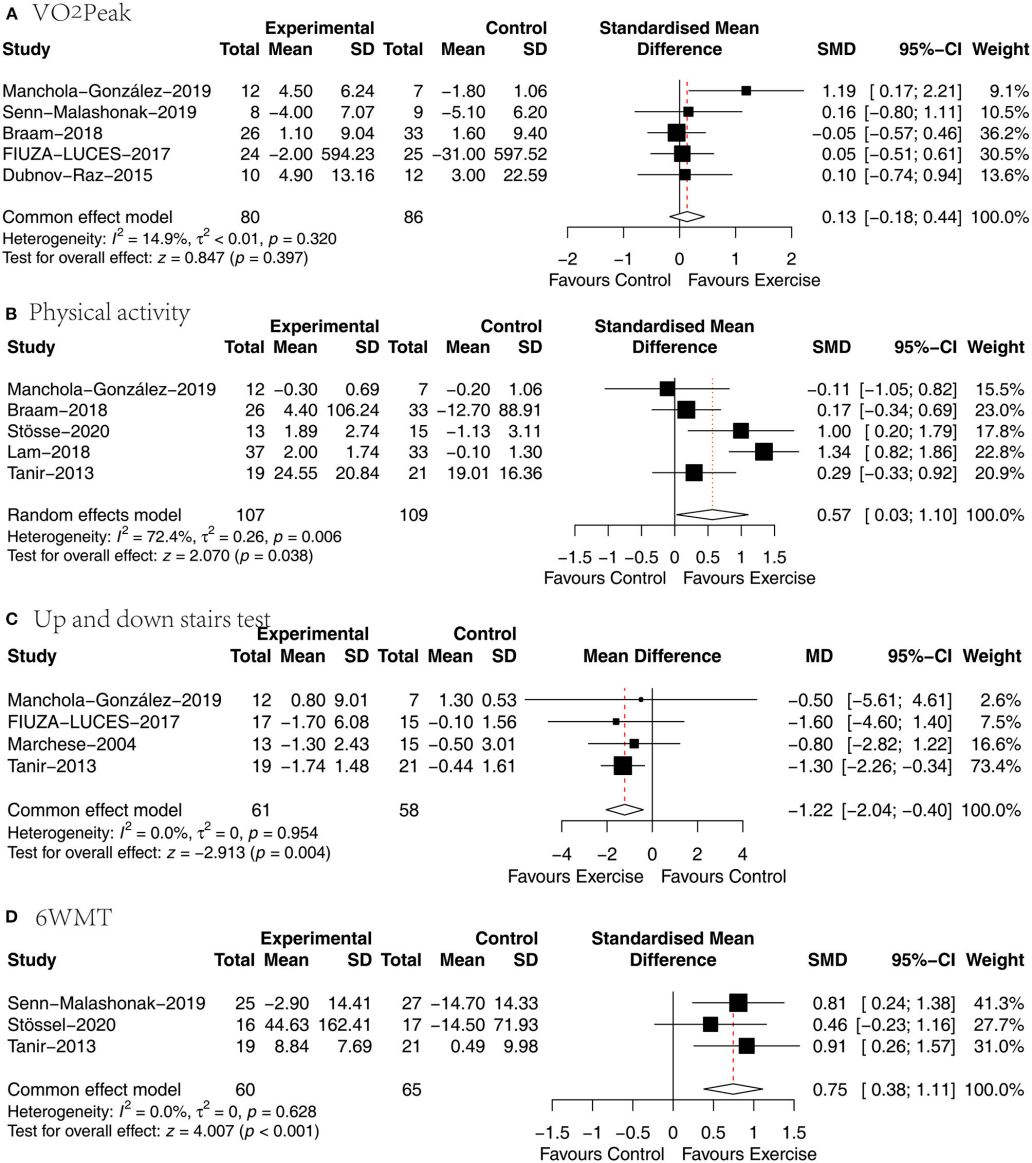
Results of meta-analysis of **(A)** VO_2_Peak, **(B)** physical activity, **(C)** up and down stairs test, **(D)** 6WMT.

Five papers (27, 29, 30, 34, 36) analyzed the effect of concurrent training on VO_2_Peak in children with malignancy, and SMD was chosen for the pooling of effect sizes because of the different measurement tools used. The heterogeneity between studies was small (*P* = 0.32, *I*^2^ = 14.9%), and a fixed-effects model was used for analysis, showing no statistically significant difference between the exercise and usual care groups in VO_2_Peak [SMD = 0.13, 95% CI (−0.18, 0.44), *P* = 0.397] (Figure 4A).

### Physical activity

Five articles (27, 31, 32, 35, 36) analyzed the effect of concurrent training on physical activity in children with malignancy, and5 the SMD was selected for effect size pooling because of the different physical activity measurement tools used. Heterogeneity between studies was significant (*P* = 0.006, *I*^2^ = 72.4%), and a random effects model was used for analysis, revealing a statistically significant difference in physical activity between the exercise and usual care groups [SMD = 0.57, 95% CI (0.03, 1.1), *P* = 0.038] (Figure 4B).

### Up and down stairs test (TUDS)

Four papers (29, 33, 35, 36) analyzed the effect of concurrent training on time spent walking up and down stairs in children with malignancy, and the WMD was selected to pool effect sizes because of the consistency of the measurement tools used. The heterogeneity between studies was small (*P* = 0.954, *I*^2^ = 0.0%), and a fixed-effects model was used for analysis, showing that the time spent walking up and down stairs was significantly shorter in the exercise group than in the usual care group [SMD = −1.22, 95% CI (−2.04, −0.4), *P* = 0.004] (Figure 4C).

### 6WMT

Three papers (30, 31, 35) analyzed the effect of concurrent training on walking distance in children with malignancy, and the SMD was chosen for the combination of effect sizes because of the different measurement times used. There was no significant heterogeneity between studies (*P* = 0.628, *I*^2^ = 0%), and a fixed-effects model was used for the analysis revealing that the exercise group walked significantly farther than the usual care group [SMD = 0.75, 95% CI (0.38, 1.11), *P* < 0.01] (Figure 4D).

### Quality of life

**Figure 5:**
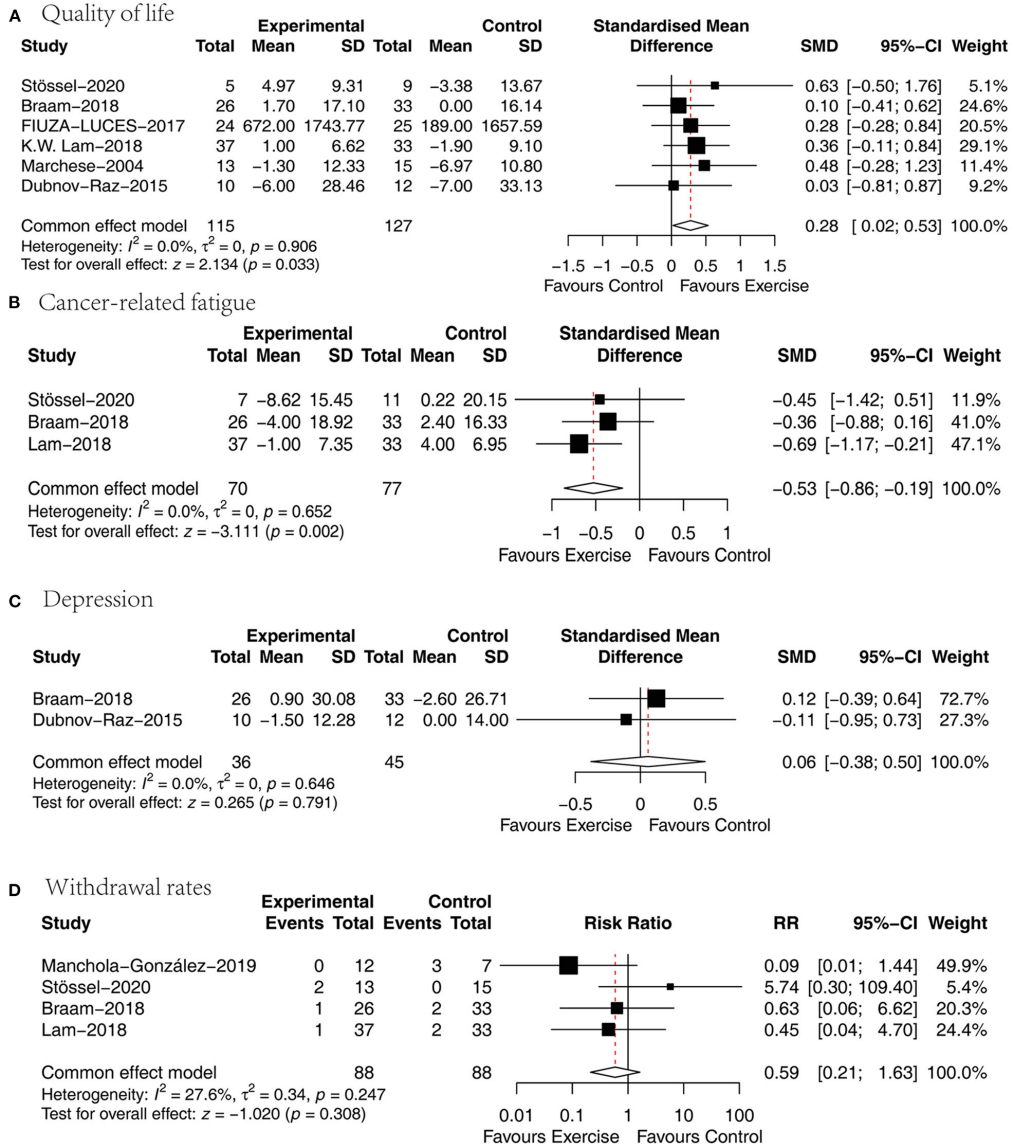
Results of meta-analysis of **(A)** quality of life, **(B)** cancer-related fatigue, **(C)** depression, **(D)** withdrawal rates.

Six papers (27, 29, 31–34) analyzed the effect of concurrent training on health-related quality of life in children with malignancy, and the SMD was selected for the pooling of effect sizes because of the different quality-of-life measurement scales used. There was no heterogeneity between studies (*P* = 0.906, *I*^2^ = 0%), and a fixed effects model was used for analysis showing a statistically significant difference between the two groups in terms of health-related quality of life [SMD = 0.28, 95% CI (0.02, 0.53), *P* = 0.033] (Figure 5A).

### Cancer-related fatigue

Three papers (27, 31, 32) analyzed the effect of concurrent training on cancer-related fatigue in children with malignancy, and the SMD was chosen for the combination of effect sizes because different cancer-caused fatigue measurement tools were used. One article used a cancer-related fatigue measurement tool that was assigned in the opposite direction to the rest of the studies, so this study data were processed according to the Cochrane Handbook so that they were assigned in the same direction. The heterogeneity between studies was small (*P* = 0.652, *I*^2^ = 0%), and a fixed-effects model was used for analysis showing that the cancer-related fatigue scores were significantly lower in the exercise group than in the usual care group [SMD = −0.53, 95% CI (−0.86, −0.19), *P* = 0.002] (Figure 5B).

### Depression

Two papers (27, 34) analyzed the effect of concurrent training on the degree of depression in children with malignancy, and the SMD was chosen for pooling effect sizes because of the different depression measurement tools. There was a small heterogeneity between studies (*P* = 0.646, *I*^2^ = 0%), and a fixed effects model was used for the analysis revealing an insignificant difference between the two groups in terms of depression [SMD = 0.06, 95% CI (−0.38, 0.5), *P* = 0.791] (Figure 5C).

### Withdrawal rates

Four papers (27, 31, 32, 36) presented active dropouts in the exercise group with those in the usual care group. There was a small heterogeneity between studies (*P* = 0.247, *I*^2^ = 27.6%), and a fixed-effects model was used for analysis showing that the difference was not statistically significant when comparing the two groups in terms of withdrawal rates [MD = 0.59, 95% CI (0.21, 1.63), *P* = 0.308] (Figure 5D).

### GRADE evidence of outcomes

The GRADE system to assess the quality of evidence of the included studies revealed low to very low quality for each outcome. This may be due to poor or missing randomization methods, allocation sequence concealment, and risk of bias due to blinding. In addition, inconsistencies may arise in the occurrence and progression of childhood malignancies. Also, the study sample sizes were inadequate, likely introducing imprecision and publication bias. Detailed information on the quality of evidence for this meta-analysis is presented in Table 2.

**Table 2:** The GRADE tool for the pooled results in the patients after concurrent training.

**Outcomes**	Illustrative comparative risks^*^(95% CI)		**Relative effect (95% CI)**	**Number of participants (studies)**	**Certainty of the evidence**
	**Assumed risk**	**Corresponding risk**			
	**Without exercise**	**With exercise**			
Muscle strength	–	Upper-limb	–	177 (4 studies)	Low^*^^†^
		The mean upper limb muscle strength in the exercise was 0.13 higher compared to usual care (−0.17 lower to 0.43 higher)			
		Lower-limb		148 (4 studies)	Low^*^^†^
		The mean lower limb muscle strength in the exercise was 0.41 higher compared to usual care (−0.08 lower to 0.74 higher)	
VO_2_ peak		The mean VO_2_peak in the exercise was 0.13 higher compared to usual care (−0.18 lower to 0.44 higher)		166 (5 studies)	Low^*^^†^
Physical activity		The mean physical activity in the exercise was 0.76 higher compared to usual care (0.2 lower to 1.33 higher)		438 (6 studies)	Very low^*^^†^^+^
TUDS		The mean TUDS in the exercise was −1.22 lower compared to usual care (−2.04 lower to −0.40 higher)		119 (4 studies)	Low^*^^†^
Walk ability		The mean walk ability in the exercise was 0.75 higher compared to usual care (0.38 lower to 1.11 higher)		125 (3 studies)	Low^*^^†^
Quality of life		The mean quality of life in the exercise was 0.19 higher compared to usual care (0.01 lower to 0.38 higher)		464 (7 studies)	Low^*^^†^
Cancer-related fatigue		The mean cancer-related fatigue in the exercise was −0.50 lower compared to usual care (−0.70 lower to −0.29 higher)		369 (4 studies)	Low^*^^†^
Depression		The mean depression in the exercise was 0.06 higher compared to usual care (−0.38 lower to 0.50 higher)		81 (2 studies)	Very low^*^^†^^‡^
Withdrawal rates	88 per 1,000	46 per 1,000	RR 0.59 (0.21, 1.63)	176 (4 studies)	Very low^*^^†^^‡^

High quality: Further research is very unlikely to change our confifidence in the estimate of effect.

Moderate quality: Further research is likely to have an important impact on our confifidence in the estimate of effect and may change the estimate.

Low quality: Further research is very likely to have an important impact on our confifidence in the estimate of effect and is likely to change the estimate.

Very low quality: We are very uncertain about the estimate.

* design limitations;

† publication bias;

‡ imprecision;

+ heterogeneity.

^*^The basis for the assumed risk (e.g., the median control group risk across studies) is provided in footnotes. The corresponding risk (and its 95% confifidence interval) is based on the assumed risk in the comparison group and the relative effect of the intervention (and its 95% CI) Control group risk estimates come from pooled estimates of control groups. Relative effect based on available case analysis.

## Discussion

Many children with malignancies experience serious health problems during and after treatment. In rehabilitation medicine, it is commonly acknowledged that exercise rehabilitation has a good effect on a patient's functional level and physical health (37). Concurrent training improves athletic performance in athletes, but this is the first study to our knowledge to examine the impact of concurrent training on physical function and quality of life in children with malignancy. This systematic review and meta-analysis of nine randomized controlled trials included 371 children with malignancies demonstrated that compared to usual care, concurrent training enhanced muscle strength, increased mobility, shortened time to and from stairs, improved walking ability, and effectively reduced fatigue in children with cancer but had no significant effect on peak oxygen uptake and depression.

The 6MWT, as a measure of the body's dynamic endurance, reflects an individual's fitness level in terms of motor endurance by measuring 6WMT (28). Currently, the 6MWT is generally used to assess the cardiopulmonary function of patients, to guide patient treatment, to reflect patient prognosis and treatment outcome, and to predict morbidity and mortality. The present meta-analysis revealed that concurrent training prolonged the 6WMT in children with malignancy, which is consistent with the findings of Morales et al. This may be because concurrent training includes aerobic exercise, a form of endurance exercise that promotes metabolism and improves cardiopulmonary function in children, thereby increasing the 6WMT. This study demonstrated that concurrent training could effectively enhance the muscle strength of the lower limbs of children. The therapy for pediatric cancer and a prolonged sedentary lifestyle leads to muscle atrophy and impacts bone health (38). Concurrent training includes resistance exercise, which effectively increases muscle strength and can stimulate skeletal muscle, increase muscle blood supply, and increase the cross-sectional area of skeletal muscle type I and type II muscle fibers, thereby preventing the reduction of muscle volume and improving muscle strength in children with the disease. The present study depicted that the concurrent training reduced the completion time and significantly improved the children's ability to walk up and down stairs, which is consistent with the physical activity outcome and may be due to the huge development of the children's muscles and endurance. Despite the excellent performance in the physical function test, no significant changes were found in some of the physical function test indicators. Concurrent training had no significant impact on peak oxygen uptake in children with malignancy, which is consistent with the findings of Braam et al. (27), Morales et al. (39) suggested that long-term monitoring of this outcome should be performed to further clarify the effectiveness of exercise.

Cancer-related fatigue (CRF) is a painful, persistent, subjective feeling of fatigue and is associated with the tumor or tumor treatment and interferes with daily life (40). Almost all patients experience CRF during oncology treatment (41), and guidelines for managing CRF have been published in the United States, Canada, the United Kingdom, and China (42, 43). These guidelines highlight exercise as a cost-effective, non-pharmacological treatment with few adverse effects. This study confirms that concurrent training has a significant ameliorative effect on fatigue in children with malignancy. The mechanism of action may be closely related to the role of regulating HPA axis dysfunction by inhibiting the release of ACTH and promoting the release of Cor. Quality of life is a common indicator of cancer treatment and is widely used to measure the health status of children with malignant tumors. This indicator is measured by the PedsQLTM scale, and this study found that concurrent training was more effective than usual care. In addition, there was no statistically significant effect of concurrent training on depression in children with malignancy, which is inconsistent with the previous studies conducted in healthy children. An RCT of children demonstrated that a 9-month physical activity intervention improved the structure and function of brain networks associated with cognitive function and had a beneficial effect on mental health (44). Exercise increases brain-derived neurological factor (BDNF) (45) in the central nervous system and increases brain dopamine, 5-hydroxytryptamine, and norepinephrine concentrations, thereby improving anxiety and depressive symptoms (46). The reasons for the lack of significant effect of concurrent training on depressive symptoms in children with malignancy may be multifaceted. The short and low frequency of the included studies with intervention periods of 12 weeks and 6 months and a frequency of 2 sessions/week failed to significantly improve depressive symptoms. Data from several large cohort studies have depicted an association between lower levels of cardiorespiratory fitness and depressive symptoms (47–49). The results of this study indicate that concurrent training did not improve VO_2_Peak in children with malignancy, which may have contributed to the failure to alleviate depressive symptoms. Hayek noted that exercise intolerance is associated with emotional distress among pediatric cancer survivors and may lead to diminished interest in exercise in children, which in turn leads to reduced training effectiveness (50). There was no significant difference in the drop-out rate between the concurrent training group and the usual care group, indicating that the concurrent training was appropriate and feasible.

Regarding the trial locations, the concurrent training interventions for children with malignant tumors were mostly conducted in developed regions and countries, while the interventions for children with malignant tumors in developing countries still faced many obstacles. For example, the primary caregivers of children with malignant tumors often questioned the safety of exercise due to conventional beliefs; there was an absence of systematic exercise intervention programs for children with malignant tumors and a lack of awareness of the importance of exercise for children with malignant tumors among the children's families and healthcare professionals. Therefore, it is recommended to provide education and guidance on exercise intervention for children and their primary caregivers, as well as to increase multidisciplinary teamwork to design a safe and scientific exercise intervention program for children with malignant tumors. Currently, research on exercise interventions for children with malignancy in developing countries is still in its infancy, so pilot studies with small samples should be conducted to investigate the feasibility and application of exercise in children with malignancy.

Finally, this study has many limitations. First, due to the specificity of the disease, there are differences in the frequency, duration, and assessment tools of exercise interventions for children with malignancy, as well as large national and regional differences in the included literature and possible differences in the ethnicity and physical quality of the children, all of which contribute to clinical heterogeneity. Second, the monitoring effect of concurrent training is not clearly stated, as the way the child performs exercise at home and afterward may affect the results. Third, given the small number of included studies, the analysis is limited, and the results may not represent the entire population. Fourth, most of the included literature does not mention allocation concealment methods and blinding, which may lead to methodological heterogeneity. Finally, the lack of adherence due to exercise modality may also impact the experimental results. For example, the negative attitudes and self-evaluations regarding the concurrent training of children in the intervention group may have influenced the accuracy of the results.

## Conclusion

Current evidence suggests that concurrent training could improve cancer-related fatigue, quality of life, muscle strength, and physical capacity in children with malignancy but has no significant effect on VO_2_Peak and Mental health. Because the quality level of evidence is mostly very low. High-quality, large multicenter randomized controlled trials are needed in the future to provide more reliable evidence.

## Data availability statement

The original contributions presented in the study are included in the article/Supplementary material, further inquiries can be directed to the corresponding author.

## Author contributions

WZ, MF, and MZ: research idea and study design. WZ, MF, HC, and HS: data acquisition. WZ and MF: data analysis/interpretation. WZ, MF, HC, JY, XH, DL, and MZ: supervision or mentorship. Each author contributed important intellectual content during manuscript drafting or revision and accepts accountability for the overall work by ensuring that questions pertaining to the accuracy or integrity of any portion of the work are appropriately investigated and resolved.
