# Supplementary material for: Effect of concurrent training on physical performance and quality of life in children with malignancy: A systematic review and meta-analysis
Source: Front Public Health. 2023 Mar 17;11:1127255. doi: 10.3389/fpubh.2023.1127255 (PMC10063894; doi:10.3389/fpubh.2023.1127255)
Supplement: Supplementary file 1 [file Data_Sheet_1.docx]

**Appendix A. PRISMA 2020 checklist.**

| **Section and Topic** | **Item #** | **Checklist item** | **Location where item is reported** |
| --- | --- | --- | --- |
| **TITLE** | | |  |
| Title | 1 | Identify the report as a systematic review. | 1 |
| **ABSTRACT** | | |  |
| Abstract | 2 | See the PRISMA 2020 for Abstracts checklist. | 1 |
| **INTRODUCTION** | | |  |
| Rationale | 3 | Describe the rationale for the review in the context of existing knowledge. | 3.4 |
| Objectives | 4 | Provide an explicit statement of the objective(s) or question(s) the review addresses. | 3.4 |
| **METHODS** | | |  |
| Eligibility criteria | 5 | Specify the inclusion and exclusion criteria for the review and how studies were grouped for the syntheses. | 4.5 |
| Information sources | 6 | Specify all databases, registers, websites, organisations, reference lists and other sources searched or consulted to identify studies. Specify the date when each source was last searched or consulted. | 4.5 |
| Search strategy | 7 | Present the full search strategies for all databases, registers and websites, including any filters and limits used. | Supplemental |
| Selection process | 8 | Specify the methods used to decide whether a study met the inclusion criteria of the review, including how many reviewers screened each record and each report retrieved, whether they worked independently, and if applicable, details of automation tools used in the process. | 4.5 |
| Data collection process | 9 | Specify the methods used to collect data from reports, including how many reviewers collected data from each report, whether they worked independently, any processes for obtaining or confirming data from study investigators, and if applicable, details of automation tools used in the process. | 4.5 |
| Data items | 10a | List and define all outcomes for which data were sought. Specify whether all results that were compatible with each outcome domain in each study were sought (e.g. for all measures, time points, analyses), and if not, the methods used to decide which results to collect. | 4.5 |
|  | 10b | List and define all other variables for which data were sought (e.g. participant and intervention characteristics, funding sources). Describe any assumptions made about any missing or unclear information. | 5 |
| Study risk of bias assessment | 11 | Specify the methods used to assess risk of bias in the included studies, including details of the tool(s) used, how many reviewers assessed each study and whether they worked independently, and if applicable, details of automation tools used in the process. | 5 |
| Effect measures | 12 | Specify for each outcome the effect measure(s) (e.g. risk ratio, mean difference) used in the synthesis or presentation of results. | 5 |
| Synthesis methods | 13a | Describe the processes used to decide which studies were eligible for each synthesis (e.g. tabulating the study intervention characteristics and comparing against the planned groups for each synthesis (item #5)). | 5 |
|  | 13b | Describe any methods required to prepare the data for presentation or synthesis, such as handling of missing summary statistics, or data conversions. | 5 |
|  | 13c | Describe any methods used to tabulate or visually display results of individual studies and syntheses. | 5 |
|  | 13d | Describe any methods used to synthesize results and provide a rationale for the choice(s). If meta-analysis was performed, describe the model(s), method(s) to identify the presence and extent of statistical heterogeneity, and software package(s) used. | 5 |
|  | 13e | Describe any methods used to explore possible causes of heterogeneity among study results (e.g. subgroup analysis, meta-regression). | 5 |
|  | 13f | Describe any sensitivity analyses conducted to assess robustness of the synthesized results. | 5 |
| Reporting bias assessment | 14 | Describe any methods used to assess risk of bias due to missing results in a synthesis (arising from reporting biases). | 5 |
| Certainty assessment | 15 | Describe any methods used to assess certainty (or confidence) in the body of evidence for an outcome. | 5 |
| **RESULTS** | | |  |
| Study selection | 16a | Describe the results of the search and selection process, from the number of records identified in the search to the number of studies included in the review, ideally using a flow diagram. | 6 |
|  | 16b | Cite studies that might appear to meet the inclusion criteria, but which were excluded, and explain why they were excluded. | 6 |
| Study characteristics | 17 | Cite each included study and present its characteristics. | 6.7 |
| Risk of bias in studies | 18 | Present assessments of risk of bias for each included study. | 7 |
| Results of individual studies | 19 | For all outcomes, present, for each study: (a) summary statistics for each group (where appropriate) and (b) an effect estimate and its precision (e.g. confidence/credible interval), ideally using structured tables or plots. | 7-11 |
| Results of syntheses | 20a | For each synthesis, briefly summarise the characteristics and risk of bias among contributing studies. | 6.7 |
|  | 20b | Present results of all statistical syntheses conducted. If meta-analysis was done, present for each the summary estimate and its precision (e.g. confidence/credible interval) and measures of statistical heterogeneity. If comparing groups, describe the direction of the effect. | 7-11 |
|  | 20c | Present results of all investigations of possible causes of heterogeneity among study results. | 7-11 |
|  | 20d | Present results of all sensitivity analyses conducted to assess the robustness of the synthesized results. |  |
| Reporting biases | 21 | Present assessments of risk of bias due to missing results (arising from reporting biases) for each synthesis assessed. | 11 |
| Certainty of evidence | 22 | Present assessments of certainty (or confidence) in the body of evidence for each outcome assessed. | 11 |
| **DISCUSSION** | | |  |
| Discussion | 23a | Provide a general interpretation of the results in the context of other evidence. | 11-15 |
|  | 23b | Discuss any limitations of the evidence included in the review. | 15 |
|  | 23c | Discuss any limitations of the review processes used. | 15 |
|  | 23d | Discuss implications of the results for practice, policy, and future research. | 14-15 |
| **OTHER INFORMATION** | | |  |
| Registration and protocol | 24a | Provide registration information for the review, including register name and registration number, or state that the review was not registered. | 3 |
|  | 24b | Indicate where the review protocol can be accessed, or state that a protocol was not prepared. | 3 |
|  | 24c | Describe and explain any amendments to information provided at registration or in the protocol. | 3 |
| Support | 25 | Describe sources of financial or non-financial support for the review, and the role of the funders or sponsors in the review. | 16 |
| Competing interests | 26 | Declare any competing interests of review authors. | 16 |
| Availability of data, code and other materials | 27 | Report which of the following are publicly available and where they can be found: template data collection forms; data extracted from included studies; data used for all analyses; analytic code; any other materials used in the review. | 1-6 |

*From:*  Page MJ, McKenzie JE, Bossuyt PM, Boutron I, Hoffmann TC, Mulrow CD, et al. The PRISMA 2020 statement: an updated guideline for reporting systematic reviews. BMJ 2021;372:n71. doi: 10.1136/bmj.n7

**Appendix B. Search strategy.**

|  | **Pubmed** |  |
| --- | --- | --- |
| **#1** | **("Neoplasms"[Mesh]) OR (((((((((((((Tumor) OR (Neoplasm)) OR (Tumors)) OR (Neoplasia)) OR (Neoplasias)) OR (Cancer)) OR (Cancers)) OR (Malignant Neoplasm)) OR (Malignancy)) OR (Malignancies)) OR (Malignant Neoplasms)) OR (Benign Neoplasms)) OR (Benign Neoplasm))** | **5,386,472** |
| **#2** | **("Exercise"[Mesh]) OR (((((((((((((Exercises) OR (Physical Activity)) OR (Physical Activities)) OR (Physical Exercise)) OR (Physical Exercises)) OR (Acute Exercise)) OR (Acute Exercises)) OR (Isometric Exercises)) OR (Isometric Exercise)) OR (Aerobic Exercise)) OR (Aerobic Exercises)) OR (Exercise Training)) OR (Exercise Trainings))** | **731,853** |
| **#3** | **("Child"[Mesh]) OR (((((child) OR (children)) OR (underage)) OR (juvenile)) OR (pediatric))** | **3,446,082** |
| **#4** | **("Controlled Clinical Trial" [Publication Type]) OR (((((Clinical Study) OR (Clinical Trial)) OR (Comparative Study)) OR (Controlled Clinical Trial)) OR (Randomized Controlled Trial))** | **3,245,923** |
| **#1 AND**  **#2 AND #3 AND #4** | **(((("Neoplasms"[Mesh]) OR (((((((((((((Tumor) OR (Neoplasm)) OR (Tumors)) OR (Neoplasia)) OR (Neoplasias)) OR (Cancer)) OR (Cancers)) OR (Malignant Neoplasm)) OR (Malignancy)) OR (Malignancies)) OR (Malignant Neoplasms)) OR (Benign Neoplasms)) OR (Benign Neoplasm))) AND (("Child"[Mesh]) OR (((((child) OR (children)) OR (underage)) OR (juvenile)) OR (pediatric)))) AND ((((((((((((((Exercises) OR (Physical Activity)) OR (Physical Activities)) OR (Physical Exercise)) OR (Physical Exercises)) OR (Acute Exercise)) OR (Acute Exercises)) OR (Isometric Exercises)) OR (Isometric Exercise)) OR (Aerobic Exercise)) OR (Aerobic Exercises)) OR (Exercise Training)) OR (Exercise Trainings)) OR ("Exercise"[Mesh]))) AND (("Controlled Clinical Trial" [Publication Type]) OR (((((Clinical Study) OR (Clinical Trial)) OR (Comparative Study)) OR (Controlled Clinical Trial)) OR (Randomized Controlled Trial)))** | **836** |

|  | **SinoMed** |  |
| --- | --- | --- |
| **#1** | **("Tumor"[Common Field:Smart] OR "Tumor"[Common Field:Smart] OR "Cancer"[Common Field:Smart] OR "Malignancy"[Common Field:Smart]) OR ("Tumor"[Unweighted:Extended])** | **6,961,210** |
| **#2** | **("Physical Activity"[Common Field:Smart] OR "Physical Activity"[Common Field:Smart] OR "Physical Activity"[Common Field:Smart] OR "Physical Activity"[Common Field:Smart] OR "Acute Exercise"[Common Field:Smart] OR "Aerobic Exercise"[Common Field:Smart] OR "Exercise Training"[Common Field:Smart] OR "Exercise Training"[Common Field:Smart] OR "Exercise"[Common Field:Smart] OR "Movement"[Common Field: Smart] OR "Resistance"[Common Field:Smart]) OR (("Motion"[Unweighted:Expanded]) OR "Exercise"[Unweighted:Expanded])** | **1,663,076** |
| **#3** | **("Child"[Common Field:Smart] OR "Child"[Common Field:Smart] OR "Minor"[Common Field:Smart] OR "Teen"[Common Field:Smart]) OR ("Child"[Unweighted:Extended])** | **2,068,132** |
| **#4** | **("Randomized controlled trial"[Common Field:Smart] OR "Randomized"[Common Field:Smart] OR "RCT"[Common Field:Smart]) OR ("Randomized controlled trial"[Unweighted:Extended])** | **2,117,768** |
| **#1 AND**  **#2 AND #3 AND #4** | **(("Child"[Common Field:Smart] OR "Child"[Common Field:Smart] OR "Minor"[Common Field:Smart] OR "Adolescent"[Common Field:Smart]) OR ("Child"[Unweighted:Expanded])) AND (("Randomized Controlled Trial"[Common Field:Smart] OR "Randomized"[Common Field:Smart] OR "RCT"[Common Field: Smart]) OR ("Randomized controlled trial"[unweighted:extended]) AND (("Physical activity"[common field:smart] OR "Physical activity"[common field:smart] OR "Physical activity"[common field:smart] OR "Physical exercise"[common field:smart] OR "Acute exercise"[common field:smart] OR "Aerobic exercise"[common field:smart] OR "Exercise training"[common field:smart] OR "Exercise Training"[Common Field:Smart] OR "Exercise"[Common Field:Smart] OR "Resistance"[Common Field:Smart]) OR (("Motion"[Unweighted:Expanded]) OR "Exercise"[Unweighted:Expanded]) AND (("Tumor"[Common Field:Smart] OR "Tumor"[Common Field:Smart ] OR "cancer"[common field:smart] OR "malignancy"[common field:smart]) OR ("tumor"[unweighted:extended]))** | **83** |

|  | **CNKI** |  |
| --- | --- | --- |
| **#1** | **(( (subject=tumor or title=tumor or v_subject=Chinese-English expansion(tumor) or title=Chinese-English expansion(tumor)) or (subject=tumor or title=tumor or v_subject=Chinese-English expansion(tumor) or title=Chinese-English expansion(tumor)) or ( (subject=Chinese-English expansion(cancer) or title=Chinese-English Expanded (Cancer) or v_subject=Cancer or title=Cancer)) or (subject=Cancer or title=Cancer or v_subject=Cancer or title=Cancer))) (fuzzy match)** | **5,962,783** |
| **#2** | **(((subject=exercise or title=exercise or v_subject=English or title=English)) or ((subject=physical activity or title=physical activity or v_subject=English or title=English)) or ((subject=aerobics or title=aerobics or v_subject=English or title=Aerobics)) or ((subject=aerobics or title=Aerobics or v_subject=English or title=Aerobics)) or ((subject=resistance exercise or title=resistance exercise or v_subject=Aerobics or title=Aerobics)) or v_subject=aerobic or v_subject=expanded in English or title=expanded in English) or (subject=resistance or v_subject=resistance or v_subject=expanded in English or title=expanded in English))) or ((subject=physical activity or v_subject=physical activity or v_subject=expanded in English or title=expanded in English))) or ((subject=physical activity or v_subject=expanded in English or title=expanded in English)) or v_subject=physical activity or v_subject=expanded (physical activity) or title=expanded (physical activity)) or (subject=acute exercise or v_subject=acute exercise or v_subject=expanded (acute exercise) or title=expanded (acute exercise)))) or ( (subject=exercise training or subject=exercise training or v_subject=expanded in English(exercise training) or title=expanded in English(exercise training)) or (subject=exercise or title=exercise or v_subject=expanded in English(exercise) or title=expanded in English(exercise)) ) ) or (subject=fitness or title=fitness or v_subject=expanded in English(exercise)) subject=fitness or title=fitness) or title=fitness)) (fuzzy match)** | **3,743,088** |
| **#3** | **((subject=children or title=children or v_subject=Children or title=Children)) or (subject=teenagers or title=teenagers or v_subject=teenagers or title=teenagers)) or (subject=children or title=children or v_subject=children or title=children)) or (subject=children or title=children or v_subject=Children or title=Children)) or (subject=Minors or title=Minors or v_subject=Minors or title=Minors)) or ( (subject=Toddlers or title=Toddlers or v_subject=Children or title=Children))) or ( (subject=Toddlers or title=Toddlers or v_subject=Children or title=Children)) extended(toddler) or title=extended(toddler)) or (subject=school-age or title=school-age or v_subject=extended(school-age) or title=extended(school-age)) ) ) (fuzzy match)** | **4,407,986** |
| **#4** | **(( (abstract=RCT or abstract_en=RCT) or (abstract=Randomized Controlled or abstract_en=Randomized Controlled)) or ( (abstract=Randomized Controlled Trial or abstract_en=Randomized Controlled Trial)) or (abstract=Randomized Controlled Trial or abstract_en=Randomized Controlled Trial)) or (abstract=Randomized Controlled Trial or abstract_en=Randomized Controlled Trial)) (exact match) Controlled Clinical Trial) or abstract_en=Controlled Clinical Trial) ) ) (exact match)** | **290,852** |
| **#1 AND**  **#2 AND #3 AND #4** | **((Subject=Children or Title=Children or v_subject=Children or title=Children)) or (Subject=Teenagers or Title=Teenagers or v_subject=Teenagers or title=Teenagers)) or (Subject=Toddlers or Title=Toddlers or v_subject=Toddlers or title=Toddlers)) or (Subject=School Age or Title=School Age or v_subject=Toddlers or title=Toddlers) or v_subject=Toddler or v_subject=Toddler or v_subject=Toddler or v_subject=Toddler or v_subject=Toddler or v_subject=Toddler or v_subject=Toddler or v_subject=Toddler or v_subject=Toddler or v_subject=Toddler or v_subject=Toddler or v_subject=Toddler or v_subject=Toddler or v_subject=Toddler or v_subject=Toddler or v_subject=Toddler or v_subject=Toddler (subject=children or title=children or v_subject=children or title=children))) and ((subject=cancer or title=cancer or v_subject=cancer or title=cancer)) and ((subject=cancer or title=cancer or v_subject=cancer or title=cancer))) and ((subject=cancer or title=cancer))) and ((subject=cancer or title=cancer))) and ((subject=cancer or v_subject=cancer or title=cancer)))) and ((subject=cancer or title=cancer))) (subject=malignant tumor or title=malignant tumor or v_subject=Middle English extension (malignant tumor) or title=Middle English extension (malignant tumor)) or ((subject=tumor or title=tumor or v_subject=Middle English extension (tumor) or title=Middle English extension (tumor)) or (subject=tumor or title=tumor or v_subject=Middle English extension (tumor)) or (subject=tumor or title=tumor or v_subject=Middle English extension (tumor)) or v_subject=tumor or v_subject=tumor or v_subject=tumor or v_subject=tumor or v_subject=tumor or v_subject=tumor or v_subject=tumor or v_subject=tumor or v_subject=tumor or v_subject=tumor or v_subject=tumor or v_subject=tumor or v_subject=tumor or v_subject=tumor) subject=Acute exercise or title=Acute exercise)))) or ((subject=Aerobic exercise or title=Aerobic exercise or v_subject=Aerobic exercise or title=Aerobic exercise)) or (subject=Exercise or title=Exercise or v_subject (subject=workout or title=workout or title=workout))) or ((subject=workout training or title=workout training or v_subject=workout training or title=workout training)) or (subject=workout or title=workout or v_subject=workout or title=workout)) or (subject=workout or title=workout or v_subject=workout) or title=Fitness)) ) ) and ((abstract=RCT or abstract_en=RCT) or (abstract=Randomized Control Time or abstract_en=RCT) ) or (abstract=Controlled Clinical or abstract_en=RCT) ) or (abstract_en=RCT) Trial) or abstract_en=Controlled Clinical Trial) ) (fuzzy match)** | **16** |

|  | **Web of Science** |  |
| --- | --- | --- |
| **#1** | **TS=（Neoplasms OR Tumor OR Neoplasm OR Tumors OR Neoplasia OR Neoplasias OR Cancer OR Cancers OR Malignancy OR Malignant Neoplasm OR Malignancies OR Malignant Neoplasms OR Benign Neoplasms OR Benign Neoplasm）** | **[8,915,675](https://www.webofscience.com/wos/alldb/summary/7e663731-abd2-4d1c-9d21-7c68ed0030dc-56364496/relevance/1)** |
| **#2** | **TS=(child OR Children OR Toddler OR Minors OR Teenagers OR School-age children OR Young people)** | **[5,097,295](https://www.webofscience.com/wos/alldb/summary/4e27d273-07e4-46af-a560-821cd225b255-5636644b/relevance/1)** |
| **#3** | **TS=(Exercises OR Physical Activity OR Physical Activities OR Physical Exercise OR Physical Exercises OR Acute Exercise OR Acute Exercises OR Isometric Exercises OR Isometric Exercise OR Aerobic Exercise OR Aerobic Exercises OR Exercise Training OR Exercise Trainings)** | **[1,909,490](https://www.webofscience.com/wos/alldb/summary/7258a428-4630-4f6d-9968-5fe9576806ff-56367b20/relevance/1)** |
| **#4** | **TS=(Clinical Study OR Clinical Trial OR Comparative Study OR Controlled Clinical Trial OR Randomized Controlled Trial)** | **[14,046,414](https://www.webofscience.com/wos/alldb/summary/33aec471-2bcf-4465-9441-bf7139c2dc31-56368bb4/relevance/1)** |
| **#5** | **#153 AND #158 AND #159 AND #161** | **[5,372](https://www.webofscience.com/wos/alldb/summary/0ca3d0de-e3f9-4d1f-a55f-cd58d17965c6-56369782/relevance/1)** |

|  | **Embase** |  |
| --- | --- | --- |
| **#1** | **'neoplasm'/exp** | **5,781,248** |
| **#2** | **'tumor':ab,ti OR 'neoplasm':ab,ti OR 'tumors':ab,ti OR 'neoplasia':ab,ti OR 'neoplasias':ab,ti OR 'cancer':ab,ti OR 'cancers':ab,ti OR 'malignant neoplasm':ab,ti OR 'malignancy':ab,ti OR 'malignancies':ab,ti OR 'malignant neoplasms':ab,ti OR 'benign neoplasms':ab,ti OR 'benign neoplasm':ab,ti** | **4,452,981** |
| **#3** | **#1 OR #2** | **6,610,635** |
| **#4** | **'child'/exp** | **3,240,234** |
| **#5** | **'child':ab,ti OR 'children':ab,ti OR 'toddler':ab,ti OR 'minors':ab,ti OR 'teenagers':ab,ti OR 'school-age children':ab,ti OR 'young people':ab,ti** | **1,915,628** |
| **#6** | **#4 OR #5** | **3,722,891** |
| **#7** | **'exercise'/exp** | **416,817** |
| **#8** | **'exercises':ab,ti OR 'physical activity':ab,ti OR 'physical activities':ab,ti OR 'physical exercise':ab,ti OR 'physical exercises':ab,ti OR 'acute exercise':ab,ti OR 'acute exercises':ab,ti OR 'isometric exercises':ab,ti OR 'isometric exercise':ab,ti OR 'aerobic exercise':ab,ti OR 'exercise training':ab,ti OR 'aerobic exercises':ab,ti OR 'exercise trainings':ab,ti** | **299,098** |
| **#9** | **#7 OR #8** | **584,472** |
| **#10** | **'exercise'/exp** | **416,871** |
| **#11** | **'clinical study':ab,ti OR 'comparative study':ab,ti OR 'clinical trial':ab,ti OR 'controlled clinical trial':ab,ti OR 'randomized controlled trial':ab,ti** | **571,527** |
| **#12** | **#10 OR #11** | **971,115** |
| **#13** | **#3 AND #6 AND #9 AND #12** | **1,685** |

|  | **Cochrane Library** |  |
| --- | --- | --- |
| **#1** | **MeSH descriptor: [Neoplasms] explode all trees** | **89835** |
| **#2** | **(Neoplasms):ab,ti,kw OR (Tumor):ab,ti,kw OR (Neoplasm):ab,ti,kw OR (Tumors):ab,ti,kw OR (Neoplasia):ab,ti,kw OR (Neoplasias):ab,ti,kw OR (Cancer):ab,ti,kw OR (Cancers):ab,ti,kw OR (Malignant Neoplasm):ab,ti,kw OR (Malignancy):ab,ti,kw OR (Malignancies):ab,ti,kw OR (Malignant Neoplasms):ab,ti,kw OR (Benign Neoplasms):ab,ti,kw OR (Benign Neoplasm):ab,ti,kw** | **236410** |
| **#3** | **#1 OR #2** | **247609** |
| **#4** | **MeSH descriptor: [Child] explode all trees** | **62000** |
| **#5** | **(child):ab,ti,kw OR (Children):ab,ti,kw OR (Toddler):ab,ti,kw OR (Minors):ab,ti,kw OR (Teenagers):ab,ti,kw OR (School-age children):ab,ti,kw OR (Young people):ab,ti,kw** | **175073** |
| **#6** | **#4 OR #5** | **175073** |
| **#7** | **MeSH descriptor: [Exercise] explode all trees** | **28782** |
| **#8** | **(Exercises):ab,ti,kw OR (Physical Activity):ab,ti,kw OR (Physical Activities):ab,ti,kw OR (Physical Exercise):ab,ti,kw OR (Physical Exercises):ab,ti,kw OR (Acute Exercise):ab,ti,kw OR (Acute Exercises):ab,ti,kw OR (Isometric Exercises):ab,ti,kw OR (Isometric Exercise):ab,ti,kw OR (Aerobic Exercise):ab,ti,kw OR (Aerobic Exercises):ab,ti,kw OR (Exercise Training):ab,ti,kw OR (Exercise Trainings):ab,ti,kw** | **118775** |
| **#9** | **#7 OR #8** | **126459** |
| **#10** | **MeSH descriptor: [Controlled Clinical Trial] explode all trees** | **127** |
| **#11** | **(Clinical Study):ab,ti,kw OR (Clinical Trial):ab,ti,kw OR (Comparative Study):ab,ti,kw OR (Controlled Clinical Trial):ab,ti,kw OR (Randomized Controlled Trial):ab,ti,kw** | **991938** |
| **#12** | **#10 OR #11** | **991938** |
| **#13** | **#3 AND #6 AND #9 AND #12** | **365** |

|  | **OVID** |  |
| --- | --- | --- |
| **#1** | **Neoplasms.mp. [mp=tx, bt, bo, ti, ed, ot, ab, ct, mc, st, or, tn, ps, ds, cb, rn, sq, mq, ge, tm, mi, sh, mx, nt, hw, id, cc, nm, fx, kf, ox, px, rx, an, ui, on, sy]** | **4518075** |
| **#2** | **(Neoplasms or Tumor or Neoplasm or Tumors or Neoplasia or Neoplasias or Cancer or Cancers or Malignancy or Malignant Neoplasm or Malignancies or Malignant Neoplasms or Benign Neoplasms or Benign Neoplasm).m_titl.** | **2876443** |
| **#3** | **#1 AND #2** | **5545133** |
| **#4** | **child.mp. [mp=tx, bt, bo, ti, ed, ot, ab, ct, mc, st, or, tn, ps, ds, cb, rn, sq, mq, ge, tm, mi, sh, mx, nt, hw, id, cc, nm, fx, kf, ox, px, rx, an, ui, on, sy]** | **3585411** |
| **#5** | **(child or Children or Toddler or Minors or Teenagers or School-age children or Young people).m_titl.** | **1235630** |
| **#6** | **#4 AND #5** | **3906901** |
| **#7** | **exercise.mp. [mp=tx, bt, bo, ti, ed, ot, ab, ct, mc, st, or, tn, ps, ds, cb, rn, sq, mq, ge, tm, mi, sh, mx, nt, hw, id, cc, nm, fx, kf, ox, px, rx, an, ui, on, sy]** | **1299695** |
| **#8** | **(Exercises or Physical Activity or Physical Activities or Physical Exercise or Physical Exercises or Acute Exercise or Acute Exercises or Isometric Exercises or Isometric Exercise or Aerobic Exercise or Aerobic Exercises or Exercise Training or Exercise Trainings).m_titl.** | **157249** |
| **#9** | **#7 AND #8** | **1351588** |
| **#10** | **Randomized Controlled Trial.mp. [mp=tx, bt, bo, ti, ed, ot, ab, ct, mc, st, or, tn, ps, ds, cb, rn, sq, mq, ge, tm, mi, sh, mx, nt, hw, id, cc, nm, fx, kf, ox, px, rx, an, ui, on, sy]** | **861946** |
| **#11** | **(Clinical Study or Clinical Trial or Comparative Study or Controlled Clinical Trial or Randomized Controlled Trial).m_titl.** | **363078** |
| **#12** | **#10 AND #11** | **1082828** |
| **#13** | **#3 AND #6 AND #9 AND #12** | **492** |

|  | **EBSCOhost** |  |
| --- | --- | --- |
| **S1** | **Neoplasms OR Tumor OR Neoplasm OR Tumors OR Neoplasia OR Neoplasias OR Cancer OR Benign Neoplasms OR Malignant Neoplasm OR Malignancy OR Malignancies OR Malignant Neoplasms** | **8,888,504** |
| **S2** | **child OR Children OR Toddler OR Minors OR Teenagers OR School-age children OR Young people** | **13,746,286** |
| **S3** | **Exercises OR Physical Activity OR Physical Activities OR Physical Exercise OR Isometric Exercise OR Aerobic Exercises OR Exercise Training OR Exercise Trainings OR Isometric Exercises OR Physical Exercises** | **2,325,763** |
| **S4** | **Clinical Study OR Clinical Trial OR Comparative Study OR Controlled Clinical Trial OR Randomized Controlled Trial** | **4,307,918** |
| **S5** | **(Clinical Study OR Clinical Trial OR Comparative Study OR Controlled Clinical Trial OR Randomized Controlled Trial) AND (S1 AND S2 AND S3 AND S4)** | **1,081** |

|  | **SPORTDiscus** |  |
| --- | --- | --- |
| **S1** | **Neoplasms OR Tumor OR Neoplasm OR Tumors OR Neoplasia OR Neoplasias OR Cancer OR Benign Neoplasms OR Malignant Neoplasm OR Malignancy OR Malignancies OR Malignant Neoplasms** | **38,109** |
| **S2** | **child OR Children OR Toddler OR Minors OR Teenagers OR School-age children OR Young people** | **158,126** |
| **S3** | **Exercises OR Physical Activity OR Physical Activities OR Physical Exercise OR Isometric Exercise OR Aerobic Exercises OR Exercise Training OR Exercise Trainings OR Isometric Exercises OR Physical Exercises** | **311,587** |
| **S4** | **Clinical Study OR Clinical Trial OR Comparative Study OR Controlled Clinical Trial OR Randomized Controlled Trial** | **93,789** |
| **S5** | **(Clinical Study OR Clinical Trial OR Comparative Study OR Controlled Clinical Trial OR Randomized Controlled Trial) AND (S1 AND S2 AND S3 AND S4)** | **74** |

|  | **Scopus** |  |
| --- | --- | --- |
| **#1** | **[neoplasms''  OR  tumor''  OR  tumors''  OR  neoplasm''  OR  neoplasia''  OR  neoplasias''  OR  cancer''  OR  cancers''  OR  malignant  AND neoplasm''  OR  malignancy''  OR  malignancies''  OR  malignant  AND neoplasms''  OR  benign  AND neoplasms  AND ''  OR  benign  AND neoplasm'']  AND  [child''  OR  children''  OR  toddler''  OR  minors''  OR  teenagers''  OR  young  AND people''  OR  school-age  AND children'']  AND  [exercises''  OR  physical  AND activity''  OR  physical  AND activities''  OR  physical  AND exercise''  OR  physical  AND exercises''  OR  acute  AND exercise''  OR  acute  AND exercises''  OR  isometric  AND exercises''  OR  isometric  AND exercise''  OR  aerobic  AND exercise''  OR  aerobic  AND exercises''  OR  exercise  AND training''  OR  exercise  AND trainings'']  AND  [clinical  AND study''  OR  clinical  AND trial''  OR  comparative  AND study''  OR  controlled  AND clinical  AND trial''  OR  randomized  AND controlled  AND trial'']** | **125** |

|  | **WANFANG DATA** |  |
| --- | --- | --- |
| **#1** | **Search expressions (subject term extensions): subject:(tumor+tumor+cancer+malignancy) and subject:(child+children+toddlers+minors+adolescents+school-age children+young adults) and subject:(physical activity+physical activity+physical exercise+acute exercise+aerobic exercise+exercise training+exercise+exercise+fitness+resistance) and abstract:(randomized controlled+ randomized controlled trial+RCT+randomized)** | **228** |

|  | **VIP** |  |
| --- | --- | --- |
| **#1** | **(((((( title or keyword=tumor OR title or keyword=tumor) OR title or keyword=cancer) OR title or keyword=malignancy) AND (((((( title or keyword=child OR title or keyword=children) OR title or keyword=young children) OR title or keyword=minors) OR title or keyword=adolescents) OR title or keyword=adolescents) OR title or keyword=school-age children) OR title or keyword=physical activity) OR title or keyword=physical activity) AND (((((( title or keyword=physical activity) OR title or keyword=physical activity) OR title or keyword=physical activity) school-age children) OR title or keyword=young adults)) AND (((((((((( title or keyword=physical activity OR title or keyword=physical activity) OR title or keyword=physical exercise) OR title or keyword=acute exercise) OR title or keyword=aerobic exercise) OR title or keyword=exercise training) OR title or keyword=exercise training) OR title or keyword=exercise) OR title or keyword=exercise) OR title or keyword=fitness) OR title or keyword=resistance)) AND (((abstract=RCT OR abstract=randomized control) OR abstract=randomized) OR abstract=experimental))** | **3** |
